# Arthroscopic Resection for Benign Fibrous Histiocytoma in the Epiphysis of the Femur

**DOI:** 10.1155/2018/8030862

**Published:** 2018-11-18

**Authors:** Junya Shimizu, Makoto Emori, Yohei Okada, Tadashi Hasegawa, Toshihiko Yamashita

**Affiliations:** ^1^Department of Orthopaedic Surgery, Sapporo Medical University School of Medicine, Japan; ^2^Department of Surgical Pathology, Sapporo Medical University School of Medicine, Japan

## Abstract

A 16-year-old woman presented to her local hospital with a few-month history of right knee pain. On MRI, the lesion exhibited an intermediate signal on T1-weighted and T2-weighted images. The orthopedic physician made a diagnosis of osteochondritis dissecans. After 13 months from the first visit, the patient underwent MRI of the knee once more, which demonstrated that the osteolytic lesion grew larger. To achieve a definitive diagnosis, we attempted to perform a resection biopsy with knee arthroscopy. We performed biopsy, and the tumor was removed completely. The tumor occurred in an epiphysis, and the pathological findings concluded that the pathological diagnosis was benign fibrous histiocytoma. One year after surgery, she was asymptomatic. Computed tomography revealed that the previous tumor bone cavity was filled with bone formation and no evidence of recurrence.

## 1. Introduction

Benign fibrous histiocytoma (BFH) is a lesion that is histologically identical to nonossifying fibroma (NOF) but occurs in the nonmetaphyseal portion of long bones or in the pelvis [1–3]. However, tumors that occur in the distal femoral epiphysis are rare. Herein, we describe the first successfully treated case of BFH in the distal lateral femoral epiphysis. Arthroscopic resection may be feasible if the benign osseous lesion occurs in the lateral epiphysis of the femur.

## 2. Case Report

**Figure 1:**
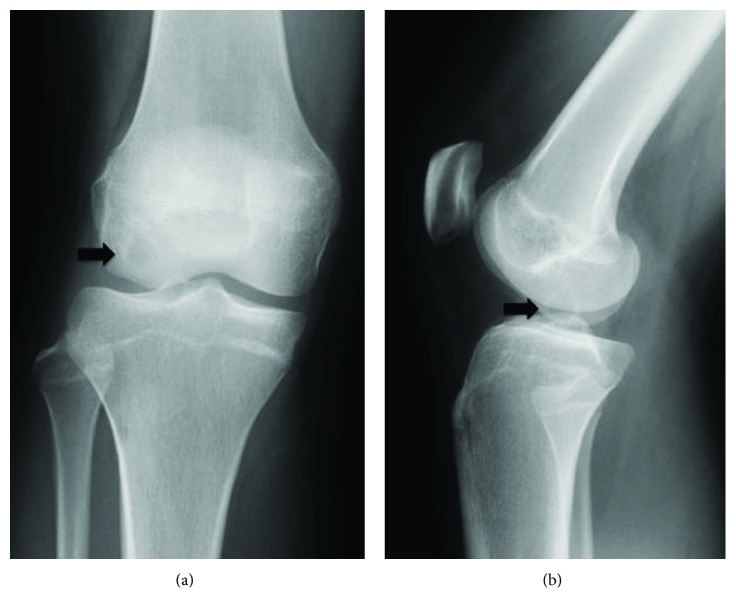
(a, b) A radiograph shows an epiphyseal osteolytic lesion in the end of the right femur (arrow).

**Figure 2:**
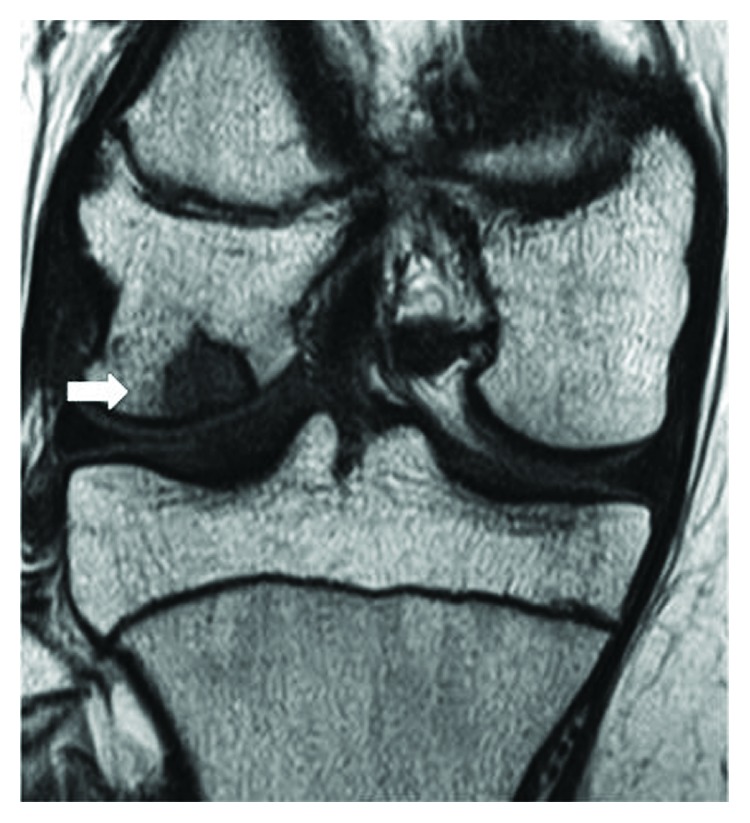
Coronal T1-weighted MR image shows a low-signal-intensity lesion (arrow).

**Figure 3:**
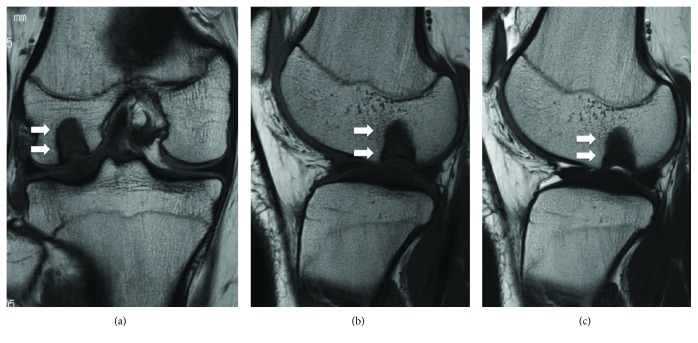
(a) Coronal T1-weighted MR image reveals the lesion growing larger. (b, c) Sagittal T1 and T2-weighted images show a low-signal-intensity lesion (arrow).

**Figure 4:**
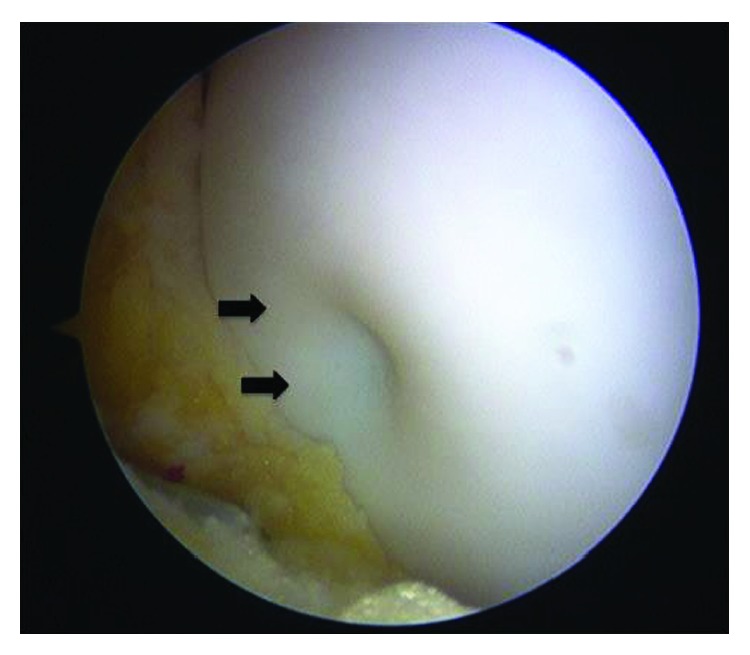
Arthroscopy showed the depressed lesion like a dimple at the lateral condyle of the femur.

**Figure 5:**
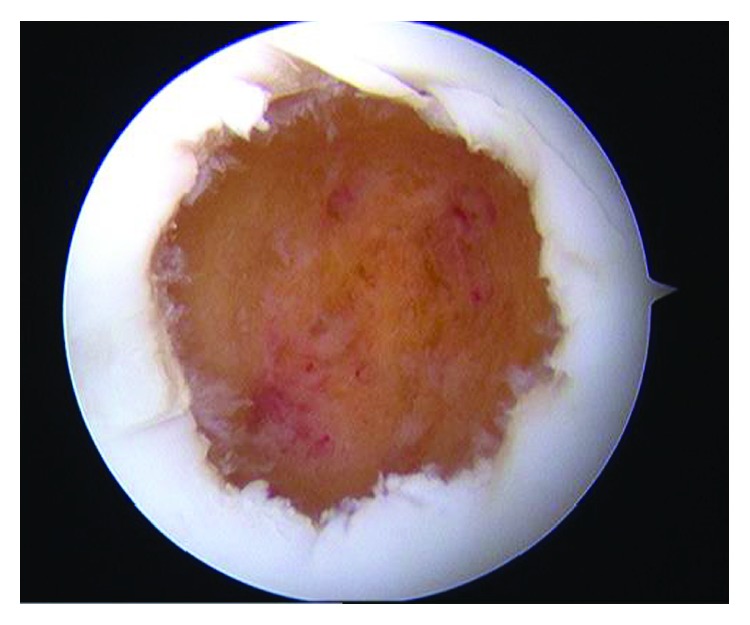
After the curettage of the tumor, the tumor was completely resected and the subchondral bone appeared to be intact.

**Figure 6:**
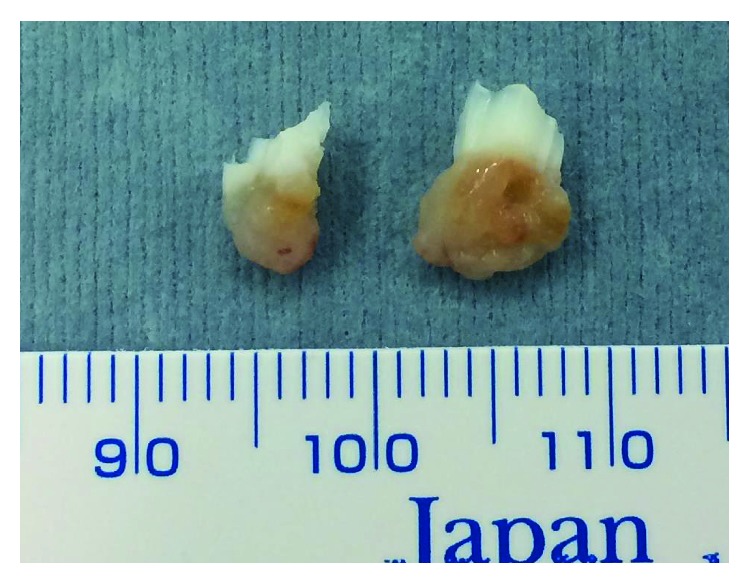
The tumor tissue was macroscopically gray-white and contains yellow brown foci.

**Figure 7:**
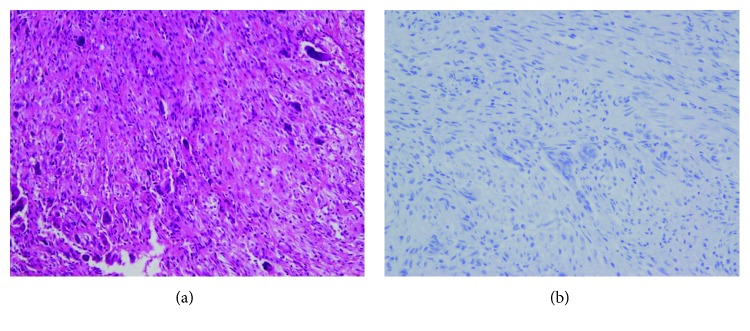
(a) Hematoxylin-eosin staining showed a storiform pattern with giant cells. (b) The tumor did not show nuclear Histone 3.3 G34W immunoreactivity.

**Figure 8:**
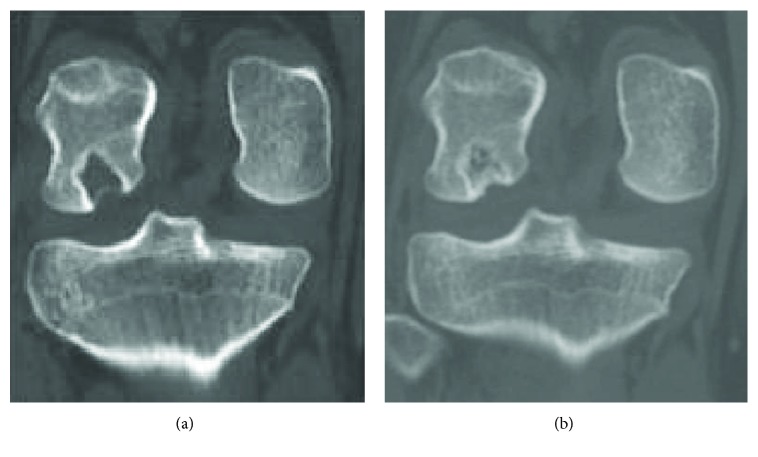
(a) Coronal computed tomography image shows a bone cavity lesion one month after the surgery. (b) Coronal computed tomography image shows the tumor bone cavity filled with bone formation one year after the surgery.

A 16-year-old woman presented to her local hospital with a few-month history of right knee pain. She was in good health and had no history of direct trauma to the knee. Upon admission, her knee motion range was not restricted and other physical examinations were nonspecific. An anteroposterior plain radiograph revealed the osteolytic lesion surrounding by the bone sclerotic lesion in the lateral femoral condyle (Figures 1(a) and 1(b)). On magnetic resonance imaging (MRI), the lesion exhibited an intermediate signal on T1-weighted (T1W) and T2-weighted (T2W) images (Figure 2). The orthopedic physician made a diagnosis of osteochondritis dissecans. After 13 months, the patient underwent MRI of the knee once more, which demonstrated that the osteolytic lesion grew larger and now measured 16 mm × 10 mm × 10 mm (Figures 3(a)–3(c)). The patient was then referred to our institution for further management. In our institution, computed tomography scans showed well-defined geographic bone destruction demarcated by the sclerotic rim. Therefore, we highly suspected the tumor of being a benign lesion, such as a fibroma. To achieve a definitive diagnosis, we attempted to perform a resection biopsy with knee arthroscopy as the epiphyseal plate did not require removal or destruction. During the surgery, anterolateral and anteromedial portals were used. Arthroscopy revealed a depressed lesion, such as the dimple at the lateral condyle of the femur (Figure 4). We performed biopsy, and the tumor was removed completely with the sharp curettes and suction during arthroscopy (Figure 5). The tumor tissue was macroscopically gray-white and contained yellow brown foci (Figure 6). The histological specimen showed a storiform pattern with giant cells (Figure 7(a)). Immunohistochemical analyses revealed that the tumor cells were negative for SOX9 and S100. The tumor did not show nuclear Histone 3.3 G34W immunoreactivity (Figure 7(b)). The tumor occurred in an epiphysis, and the pathological findings concluded that the pathological diagnosis was BFH. Full weight-bearing was allowed soon after surgery as the tumor was located in the lateral condyle of the femur. One year after surgery, she was asymptomatic. Computed tomography revealed that the previous tumor bone cavity was filled with bone formation and no evidence of recurrence (Figure 8).

## 3. Discussion

Bone tumors have a predilection for specific bones and specific sites in bones. Different diagnoses for bone tumors that develop in the epiphysis are mostly benign lesions, such as chondroblastoma, giant cell tumor of bone (GCTB), and intraosseous ganglion. Only clear cell chondrosarcoma is a differential diagnosis in malignant bone tumors. In such cases, the tumor exhibits intra-articular geographic bone destruction in the distal femoral epiphysis and shares a sharply demarcated margin with the sclerotic rim. We considered that the tumor might be a chondroblastoma. Several authors have reported cases of chondroblastoma arising from the end of the femurs that are treated with arthroscopy [4, 5]. Therefore, we performed an arthroscopic procedure that included biopsy and curettage.

BFH of the bone is rare, with approximately 40% occurring in the long bones. The most common sites of BFH of the bone include the femur and tibia. BFH grows relatively slow, and the common age of incidence is in the third and fourth decades of life. Previous reports have stated that NOF is pathologically extremely similar to BFH [2, 6, 7]. However, the most common site of occurrence differs between the two conditions. Whereas NOF usually arises from the metaphysis of long bones, BFH often arises from the epiphysis. Interestingly, in this case, the histology of the tumor includes spindle-shaped fibroblasts that are arranged in a storiform pattern, which is similar to NOF. We concluded that the tumor was of the BFH type, on the basis of immunohistochemical analysis of SOX9, S100, and Histone 3.3 G34W. This was done to distinguish the lesion in this case from other bone tumors, such as GCTB and chondroblastoma.

Ultimately, we found that arthroscopic resection of a benign lesion was a useful form of treatment in this case because full weight-bearing was allowed soon after surgery. However, such a procedure was useful in this case as (1) the tumor was in the lateral epiphysis of the distal femur, (2) the tumor was nearly less than 10 mm in diameter, (3) the tumor did not destroy the epiphyseal plate, and (4) the tumor was not a chondroblastoma and so aggressive curettage was not necessary. However, arthroscopic procedures have some limitations. First, there is a risk of intra-articular spread if the tumor is malignant. In addition, adequate visualization of the tumor may not always be possible and bone grafting of the tumor cavity cannot be performed through the arthroscope. Finally, curettage of epiphyseal bone tumors may damage the articular cartilage, thereby resulting in osteoarthritis [8]. However, biopsy and treatment for benign bone tumors through arthroscopy can minimize damage to cartilage and prevent osteoarthritis.

## 4. Conclusions

Arthroscopic resection may be an attractive procedure if it is likely that the lesion of interest in the lateral end of the distal femur is a benign tumor.
